# An unexpected link between fatty acid synthase and cholesterol synthesis in proinflammatory macrophage activation

**DOI:** 10.1074/jbc.RA118.001921

**Published:** 2018-02-20

**Authors:** Richard G. Carroll, Zbigniew Zasłona, Silvia Galván-Peña, Emma L. Koppe, Daniel C. Sévin, Stefano Angiari, Martha Triantafilou, Kathy Triantafilou, Louise K. Modis, Luke A. O'Neill

**Affiliations:** From the ‡ School of Biochemistry and Immunology, Trinity Biomedical Science Institute, Trinity College, Dublin 2, Ireland,; the § Immunology Catalyst, GlaxoSmithKline, Gunnels Wood Road, Stevenage SG1 2NY, United Kingdom,; the ‖ Institute of Infection and Immunity, School of Medicine, University Hospital of Wales, Cardiff University, Cardiff CF14 4XW, Wales, United Kingdom, and; ¶ Cellzome, GlaxoSmithKline, Meyerhofstrasse 1, Heidelberg 69117, Germany

**Keywords:** fatty acid synthase (FAS), cholesterol regulation, Toll-like receptor (TLR), lipid raft, innate immunity, inflammation, cytokine, macrophage, Toll-like receptor 4 (TLR4)

## Abstract

Different immune activation states require distinct metabolic features and activities in immune cells. For instance, inhibition of fatty acid synthase (FASN), which catalyzes the synthesis of long-chain fatty acids, prevents the proinflammatory response in macrophages; however, the precise role of this enzyme in this response remains poorly defined. Consistent with previous studies, we found here that FASN is essential for lipopolysaccharide-induced, Toll-like receptor (TLR)-mediated macrophage activation. Interestingly, only agents that block FASN upstream of acetoacetyl-CoA synthesis, including the well-characterized FASN inhibitor C75, inhibited TLR4 signaling, while those acting downstream had no effect. We found that acetoacetyl-CoA could overcome C75's inhibitory effect, whereas other FASN metabolites, including palmitate, did not prevent C75-mediated inhibition. This suggested an unexpected role for acetoacetyl-CoA in inflammation that is independent of its role in palmitate synthesis. Our evidence further suggested that acetoacetyl-CoA arising from FASN activity promotes cholesterol production, indicating a surprising link between fatty acid synthesis and cholesterol synthesis. We further demonstrate that this process is required for TLR4 to enter lipid rafts and facilitate TLR4 signaling. In conclusion, we have uncovered an unexpected link between FASN and cholesterol synthesis that appears to be required for TLR signal transduction and proinflammatory macrophage activation.

## Introduction

In immune cells, different activation states require distinct types of metabolism for the cell to carry out their biological function. For example, in response to the Gram-negative bacterial product, LPS, macrophages switch their core metabolism from oxidative phosphorylation to glycolysis to control the production of ATP (1). This is a prerequisite to promote inflammation and fight infection as inhibitors of glycolysis reduce macrophage activation (2, 3). Conversely, macrophages that play a key role in wound healing and tissue repair meet their energetic requirements predominantly using oxidative phosphorylation (4).

Whereas alterations in glucose metabolism have been well-characterized, it is now evident that lipid metabolism is also altered depending on the activation state of a cell (5). The consumption of fatty acids through β-oxidation in the mitochondria is the predominant fuel used by M2 or alternatively activated macrophages, and this promotes an anti-inflammatory phenotype (6, 7). On the other hand, synthesis of lipids is associated with a pro-inflammatory phenotype (2, 8). Whereas inhibition of glycolysis prevents a pro-inflammatory response, there have only been limited studies on whether lipid metabolism is also essential for the pro-inflammatory response or whether it may have the potential to be targeted therapeutically.

Fatty acid synthase (FASN)^2^ is a homodimeric enzyme that contains multiple catalytic domains that operate in a cyclical loop to produce palmitate. The precursor metabolites used for palmitate synthesis are acetyl-CoA and malonyl-CoA with the carbon backbone being extended by 2 carbons with each cycle through a series of six intermediate reactions. First, acetyl-CoA and malonyl-CoA are conjugated to the acyl-carrier protein (ACP) by the malonyl/acetyltransferase reversible enzymatic domain, and this allows the retention of the metabolite to FASN. Acetyl-ACP and malonyl-ACP then undergo a condensation reaction to produce acetoacetyl-ACP in the 3-ketoacyl synthase domain. Acetoacetyl-ACP is reduced to β-hydroxybutyryl-ACP in the 3-ketoacyl reductase domain, dehydrated to crotonyl-ACP by the 3-hydroxyacyl dehydrase domain, and finally reduced to butyryl-ACP by the enoyl reductase domain. As this cycle repeats, a new malonyl-CoA molecule is used to extend the backbone by 2 carbons until a 16-carbon chain of palmitate is produced and is cleaved by the thioesterase domain (9, 10).

FASN is predominantly regulated by the sterol regulatory element–binding proteins (SREBPs), of which there are three isoforms: SREBP1a, SREBP1c, and SREBP2 (11). Whereas SREBP1 has been more associated with transcription of fatty acid synthesis genes and SREBP2 more associated with cholesterol-related genes, they are both activated by the same signal, a reduction in cholesterol. This indicates an uncharacterized link between fatty acid synthesis and cholesterol synthesis.

The synthesis of cholesterol in cells is a complex pathway that uses acetyl-CoA to produce the 27-carbon structure of cholesterol (12). Cholesterol provides structure and fluidity to the membranes of the cell and allows mammalian cells to dispense with a cell wall. Similar to fatty acid synthesis, low levels of cellular cholesterol activate the SREBPs to induce a range of genes associated with cholesterol production and cholesterol uptake. Conversely, when cholesterol levels are high, they activate the liver X receptor transcription factors that promote the transcription of cholesterol exporters to balance cholesterol levels via cholesterol efflux (13, 14). It is important for the cell to be capable of tightly regulating cholesterol levels. For instance, high levels of cholesterol in macrophages lead to foam cell differentiation, and these cholesterol-rich macrophages are associated with a poor prognosis in atherosclerosis (15, 16), demonstrating that cholesterol metabolism is a key biosynthetic pathway in macrophages.

Here we provide evidence for a biosynthetic link between fatty acid synthase and cholesterol synthesis. Our data suggest that the production of acetoacetyl-CoA by FASN through its ketoacyl synthase domain during LPS stimulation is important for a pro-inflammatory response. The mechanism involves a link to cholesterol synthesis from FASN-derived acetoacetyl-CoA that is required for cholesterol synthesis and subsequent lipid raft formation, which is essential for TLR signaling. We conclude that the synthesis of cholesterol during LPS stimulation is closely tied to FASN, identifying a previously unknown link between these two biosynthetic link pathways.

## Results

### FASN is essential for inflammatory signaling in response to LPS

**Figure 1.**
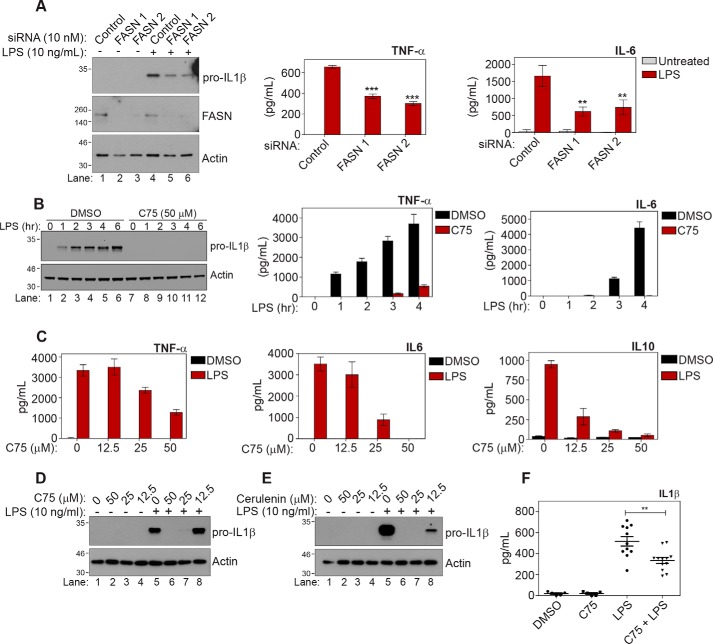
**Fatty acid synthase is required for a pro-inflammatory response to LPS.** *A*, BMDMs were transfected with either a scramble siRNA control or two independent FASN siRNAs (10 nm) for 96 h. Cells were then treated with LPS (10 ng/ml) for 4 h. IL1β was measured by Western blotting. TNF-α, IL6, and IL10 were measured by ELISA. *B*, BMDMs were pretreated with DMSO or C75 (50 μm) for 1 h. Cells were then treated with LPS (10 ng/ml) for the indicated times. *C–E*, BMDMs were pretreated with the indicated dose of C75 (*C* and *D*) or cerulenin (*E*) for 1 h. Cells were then treated with LPS (10 ng/ml) for 4 h. *F*, C57Bl mice were injected intraperitoneally with vehicle or C75 (10 mg/kg) for 30 min. Mice were subsequently injected intraperitoneally with LPS (10 mg/kg) for 90 min. Serum was isolated from whole blood, and levels of IL1β, TNF-α, and IL10 were measured by ELISA. Western blots are representative of three independent experiments. *A*, *B*, and *E*, graphs represent the mean ± S.E. (*error bars*) (*n* = 3). *A* and *F*, data represent mean ± S.E. (*n* = 12/group); *, *p* < 0.05; **, *p* < 0.01; ***, *p* < 0.001, one-way ANOVA.

To investigate the role of FASN in macrophage activation, we first used a genetic approach to knock down the FASN protein using two independent siRNAs. We found that following reduction of FASN protein in macrophages, they produced less IL1β, TNF-α, and IL6 in response to LPS stimulation (Fig. 1*A*). To further establish a role for FASN in the LPS activation of macrophages by LPS, we used the well-characterized FASN inhibitor, C75. As can be seen in Fig. 1*B*, C75 effectively inhibited the production of the pro-inflammatory cytokines IL1β and TNF-α and the anti-inflammatory cytokine IL10 at all time points tested following LPS stimulation. Furthermore, we found that FASN inhibition led to a decrease in TNF-α, IL6, IL10 (Fig. 1*C*), and IL1β (Fig. 1*D*, *lanes 6* and *7* compared with *lane 5*) in a dose-dependent manner upon LPS stimulation. A second characterized inhibitor of FASN, cerulenin, also had a similar effect, as it decreased IL1β induction in response to LPS in a dose-dependent manner (Fig. 1*E*, *lanes 6* and *7* compared with *lane 5*). Neither inhibitor exerted any cytotoxic effects in bone marrow–derived macrophages (BMDMs) (Fig. S1*A*). Finally, we also investigated whether FASN inhibition *in vivo* had similar effects and found that C75 significantly reduced serum IL1β levels in response to LPS (Fig. 1*F*). These results indicate that FASN activity, *in vitro* and *in vivo*, is required for macrophage activation and IL1β production in response to LPS.

### FASN is essential for a variety of inflammatory mediators

**Figure 2.**
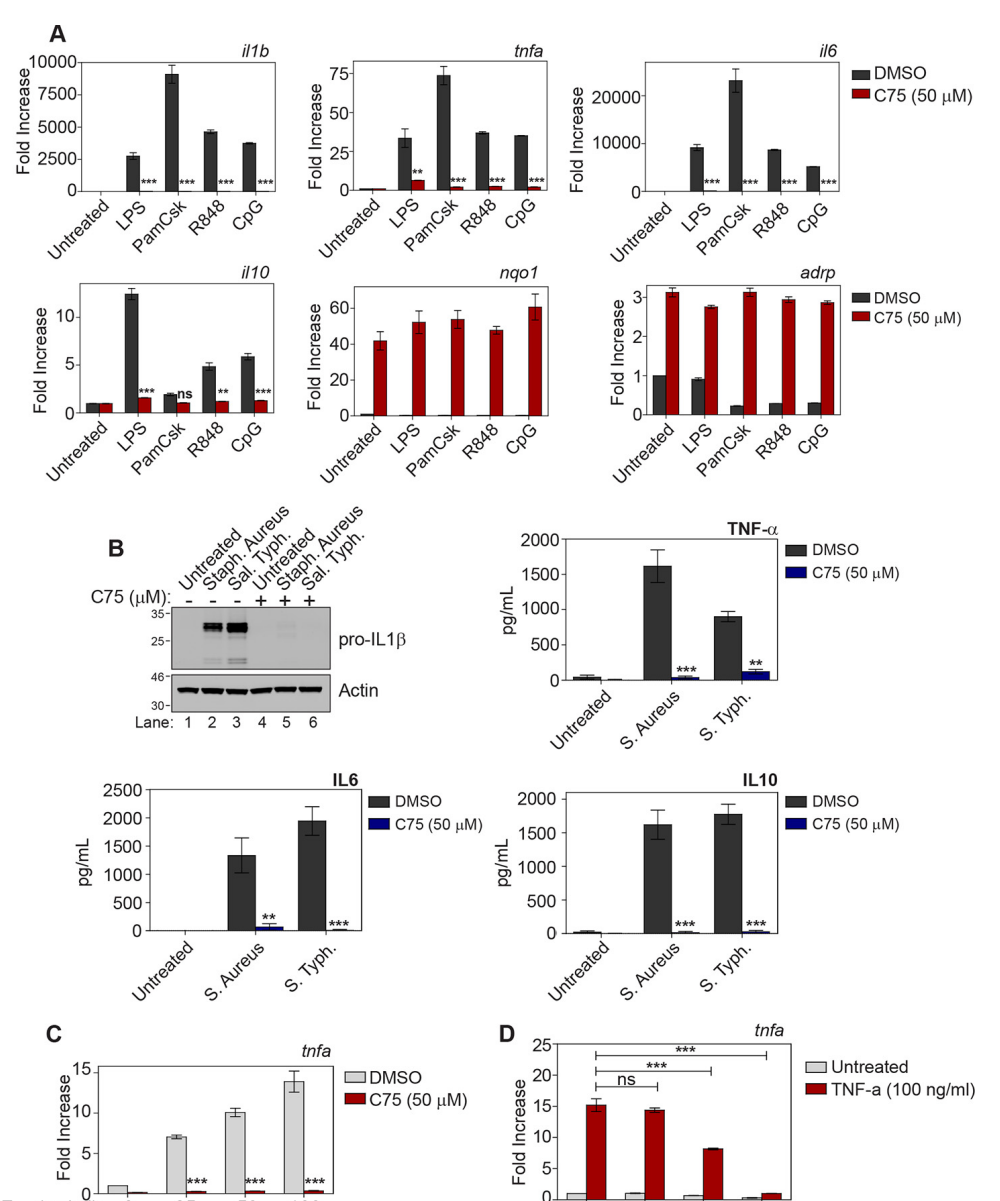
**Fatty acid synthase is essential for response to various inflammatory stimuli.** *A*, BMDMs were pretreated with DMSO or C75 (50 μm) for 1 h. Cells were then treated with LPS (10 ng/ml), Pam(3)Csk(4) (1 μg/ml), R848 (1 μg/ml), or CpG DNA (1 μg/ml) for 4 h. mRNA was extracted from whole-cell lysate and analyzed by qPCR for the indicated genes. *B*, BMDMs were pretreated with DMSO or C75 (50 μm) for 1 h. Subsequently, cells were treated with heat-killed *S. aureus* (10^8^ cells/ml) or heat-killed *S. typhimurium* (10^9^ cells/ml) for 4 h. IL1β was measured by Western blotting. TNF-α, IL6, and IL10 were measured by ELISA. *C* and *D*, BMDMs were pretreated with DMSO or the indicated dose of C75 for 1 h. BMDMs were subsequently treated with the indicated dose of TNF-α for 4 h. mRNA was extracted, and levels of *Tnfa* were analyzed by qPCR. *A–C*, graphs represent the mean ± S.E. (*error bars*) (*n* = 3). **, *p* < 0.01; ***, *p* < 0.001; *ns*, not significant, one-way ANOVA.

We next expanded our study to investigate whether FASN was a key regulator for other activators of macrophages. As demonstrated in Fig. 2*A*, FASN inhibition dramatically reduced the levels of *Il1b*, *Tnfa*, *Il6*, and *Il10* in response to a broad range of TLR agonists (LPS, Pam3Csk4, R848, and CpG DNA). C75 induced the expression of two genes that have been reported to increase with C75 treatment: the adipose-related gene, *Adrp*, and the antioxidant gene, *Nqo1*, demonstrating that inhibition of FASN did not merely lead to an overall inhibition of cellular transcription (17, 18). The TLR ligands had no effect on the gene responses of *Adrp* or *Nqo1*. As shown in Fig. 2*B*, FASN inhibition also reduced the levels of IL1β, TNF-α, IL6, and IL10 in response to both heat-killed *Staphlyococcus aureus* and *Salmonella typhurium*. C75 also inhibited induction of *Tnfa* mRNA in response to a range of doses of TNF-α itself (Fig. 2*C*). The effect of FASN inhibition in TNF-α activated BMDMs also occurred in a dose-dependent manner (Fig. 2*D*). This meant that the biological effect of C75 was common to both TLR and TNF-α signaling pathways. Altogether, these results show that FASN plays a broad regulatory role in response to a wide variety of inflammatory stimuli in macrophages.

### The ketoacyl synthase domain of FASN is essential for macrophage activation

**Figure 3.**
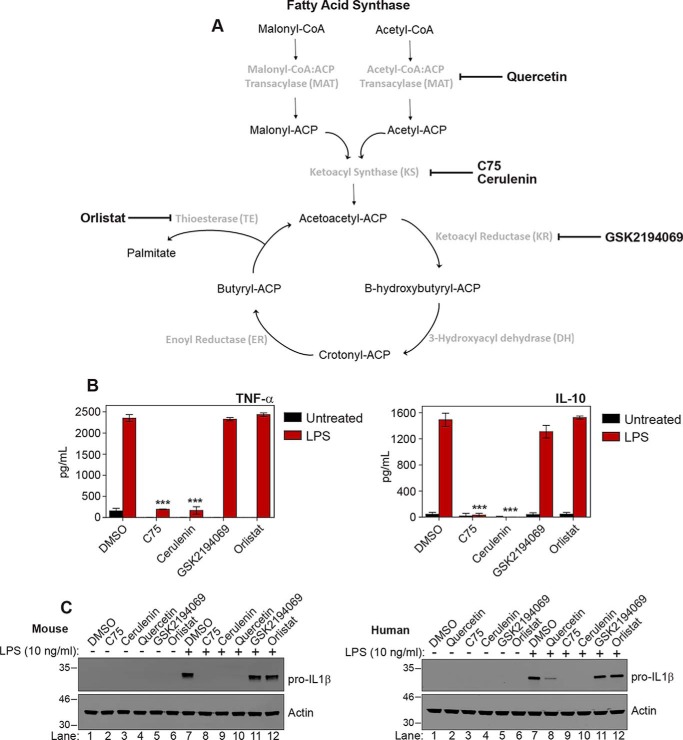
**The ketoacyl synthase domain of FASN is essential for macrophage activation.** *A*, schematic representation of reactions carried out by the holoenzyme of FASN to produce palmitate. FASN domains and inhibitors used to target them are in *boldface type. B* and *C*, BMDMs or human monocyte–derived macrophages were pretreated with C75 (50 μm), cerulenin (50 μm), GSK2194069 (1 μm), or orlistat (100 μm in mice and 50 μm in humans) for 1 h. Cells were subsequently treated with LPS (10 ng/ml) for 4 h. IL1β was measured by Western blotting. TNF-α and IL10 were measured by ELISA. Graphs represent the mean ± S.E. (*error bars*) (*n* = 4). ***, *p* < 0.001, one-way ANOVA.

FASN is a multidomain enzyme that, through a series of cyclical reactions, extends a carbon backbone for the production of palmitate (10). To investigate whether the six different enzymatic domains of FASN had varying roles in the response to LPS, we treated BMDMs with a range of known FASN inhibitors that targeted different domains (Fig. 3*A*). Fig. 3*B* illustrates the effect of the FASN inhibitors IL1β, TNF-α, and IL10 production. We found that inhibition at or before the ketoacyl synthase domain (with quercetin, cerulenin, or C75) prevented the induction of TNF-α or IL1β LPS stimulation (Fig. 3, *B* and *C*, *lanes 8–10*). However, inhibitors that targeted enzymatic domains downstream of the ketoacyl synthase domain (GSK2194069 and orlistat) had no effect on LPS-mediated effects in either mouse BMDMs or human monocyte–derived macrophages (Fig. 3 (*B* and *C*), *lanes 11* and *12*). We concluded that enzymatic domains of FASN that were at or above the ketoacyl synthase domain were critical for LPS activation of macrophages, whereas other domains within the FASN holoenzyme were dispensable.

### Acetoacetyl-CoA is a key metabolite involved in C75 inhibition of macrophage activation

**Figure 4.**
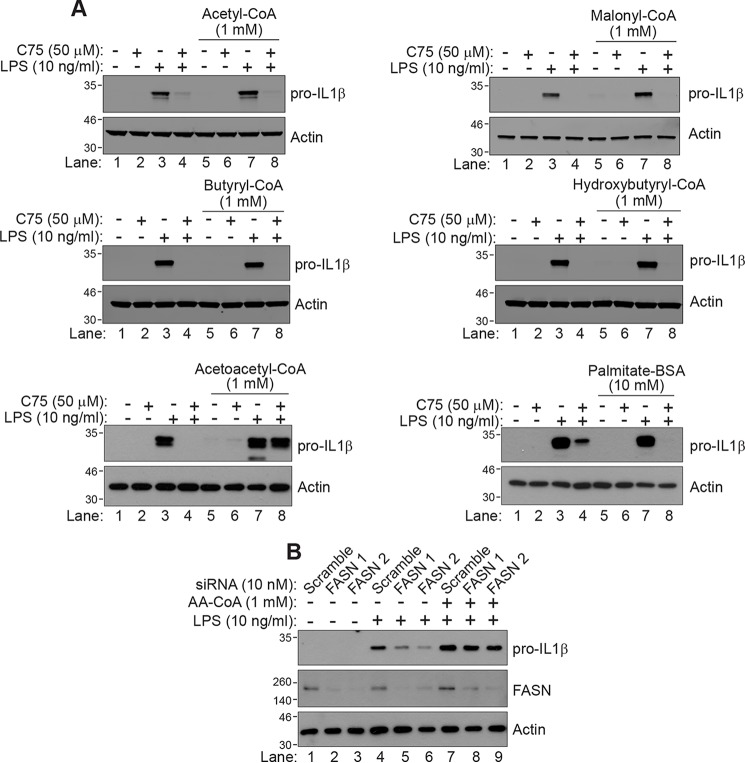
**Acetoacetyl-CoA is a key metabolite involved in C75 inhibition of macrophage activation.** *A*, BMDMs were cultured in medium containing the intermediate metabolites of fatty acid synthase: acetyl-CoA (1 mm), malonyl-CoA (1 mm), butyryl-CoA (1 mm), hydroxybutyryl-CoA (1 mm), acetoacetyl-CoA (1 mm), or palmitate-BSA (10 mm) for 3 h before treatment. Cells were then treated with DMSO or C75 (50 μm) for 1 h, followed by LPS (10 ng/ml) treatment for 4 h. IL1β was measured by Western blotting. *B*, macrophages were treated with either control siRNA or two independent siRNAs (10 nm) targeting FASN for 96 h. Cells were then pretreated with acetoacetyl-CoA (1 mm) for 3 h, followed by treatment with LPS (10 ng/ml) for 4 h. IL1β and FASN levels were measured by Western blotting.

Following the observation that different enzymatic domains of FASN had varying effects on LPS signaling, we hypothesized that intermediate metabolites produced by different FASN domains could be contributing to the cellular responses of LPS, perhaps even independently of their role in palmitate synthesis. To further investigate the role of FASN intermediate metabolites, we supplemented the medium with each of the intermediate metabolites (acetyl-CoA, malonyl-CoA, acetoacetyl-CoA, butyryl-CoA, hydroxybutyryl-CoA, and palmitate) in BMDMs activated with LPS and C75. This is a common approach used to study inhibitors of FASN, as the metabolites are stable in solution for up to 24 h (9, 19, 20). Interestingly, only one intermediate metabolite prevented the inhibition of IL1β during FASN inhibition, acetoacetyl-CoA. This can be seen in Fig. 4*A*, where, in the case of acetyl-CoA, malonyl-CoA, butyryl-CoA, hydroxybutyryl-CoA, or palmitate, no modulation of C75 effects was seen (compare *lane 8* with *lane 4* in each case), whereas acetoacetyl-CoA blocked the inhibitory effect of C75 (*bottom left panel*; compare *lane 8* with *lane 4*). This result was interesting, as acetoacetyl-CoA is produced by the ketoacyl synthase domain, the same domain that the inhibitor panel screen indicated was critical for macrophage activation. We next confirmed that acetoacetyl-CoA could be taken up by macrophages. Although metabolomic analysis did not detect acetoacetyl-CoA itself, most likely due to the extraction protocol used, it showed a significant increase in coenzyme A metabolism suggestive of uptake of acetoacetyl-CoA (Fig. S2, *A* and *B*). We next tested whether the reduction of IL1β in response to LPS following FASN knockdown could be prevented by acetoacetyl-CoA. We found that acetoacetyl-CoA could prevent IL1β reduction following FASN knockdown, further supporting our hypothesis (Fig. 4*B*, compare *lanes 8* and *9* with *lanes 5* and *6*). These data indicate that there is a surprising link between the metabolite acetoacetyl-CoA produced in fatty acid synthesis and LPS action in macrophages.

### LPS signaling is abrogated following FASN inhibition

**Figure 5.**
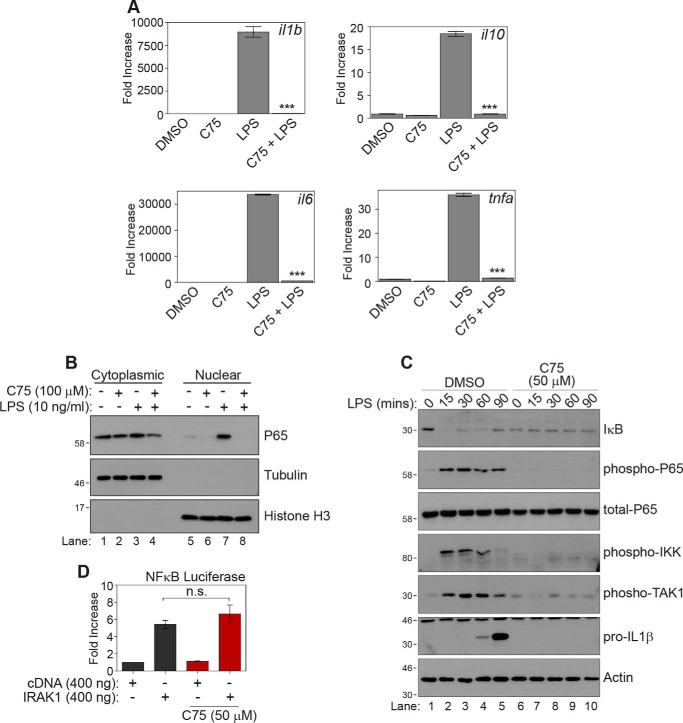
**Fatty acid synthase inhibition blocks all associated LPS signaling.** *A*, BMDMs were treated with DMSO or C75 (50 μm) for 1 h. Cells were then treated with LPS (10 ng/ml) for 4 h. mRNA was extracted from whole-cell lysate, and the indicated genes were analyzed by qPCR. Graphs represent the mean ± S.E. (*error bars*) (*n* = 3). *B*, BMDMs were treated with DMSO or C75 (50 μm) for 1 h. Cells were then treated with LPS for 30 min. Nuclear and cytoplasmic fractions were prepared from the cell lysate, and the location of p65 was analyzed by Western blotting. *C*, BMDMs were treated with DMSO or C75 (50 μm) for 1 h. Cells were then treated with LPS (10 ng/ml) over a 90-min time course at the times indicated. Phosphorylation of p65, IKKα/β, and TAK1 of the TLR4 pathway was analyzed by Western blotting. *D*, 293T cells were transfected with an NFκB luciferase construct (250 ng) and TK *Renilla* construct (150 ng) along with empty vector or IRAK1 cDNA (1 μg). Cells were treated with C75 (50 μm for 4 h). NFκB activated was measured using luciferin, whereas TK *Renilla* acted as a control with coelantrazine. *n.s.*, not significant, one-way ANOVA.

We next wanted to establish at which point of the TLR4 pathway acetoacetyl-CoA might be exerting its effects. First we wished to find whether the inhibition of LPS-mediated cytokine production occurred at the pre- or post-transcriptional level. To this end, we analyzed mRNA levels of pro-inflammatory cytokines in BMDMs that had been treated with LPS and C75. We found that FASN inhibition decreased *Il1*β, *Tnfa*, *Il6*, and *Il10* at the transcriptional level (Fig. 5*A*). We found that FASN inhibition also led to a dramatic reduction in p65 translocation to the nucleus following LPS stimulation (Fig. 5*B*, compare *lane 8* with *lane 7*), demonstrating that the inhibition was occurring upstream of p65 nuclear translocation. As shown in Fig. 5*C*, FASN inhibition also prevented the degradation of IκB and dramatically reduced the phosphorylation of p65, IKKα/β, and TAK1 while also blocking the production of IL1β (compare *lanes 6–10* with *lanes 1–5*). This result suggested that the inhibition of the TLR4 pathway by C75 was upstream in the pathway and proximal to the receptor. Furthermore, C75 had no effect on TLR4 activation that was independent of LPS. As can be seen in Fig. 5*D*, C75 had no effect on NFκB activation when the TLR4 pathway was activated by overexpressing IRAK1, further suggesting that C75 was not nonspecifically blocking components of the TLR4 pathway.

### FASN produces metabolites to promote the synthesis of cholesterol

**Figure 6.**
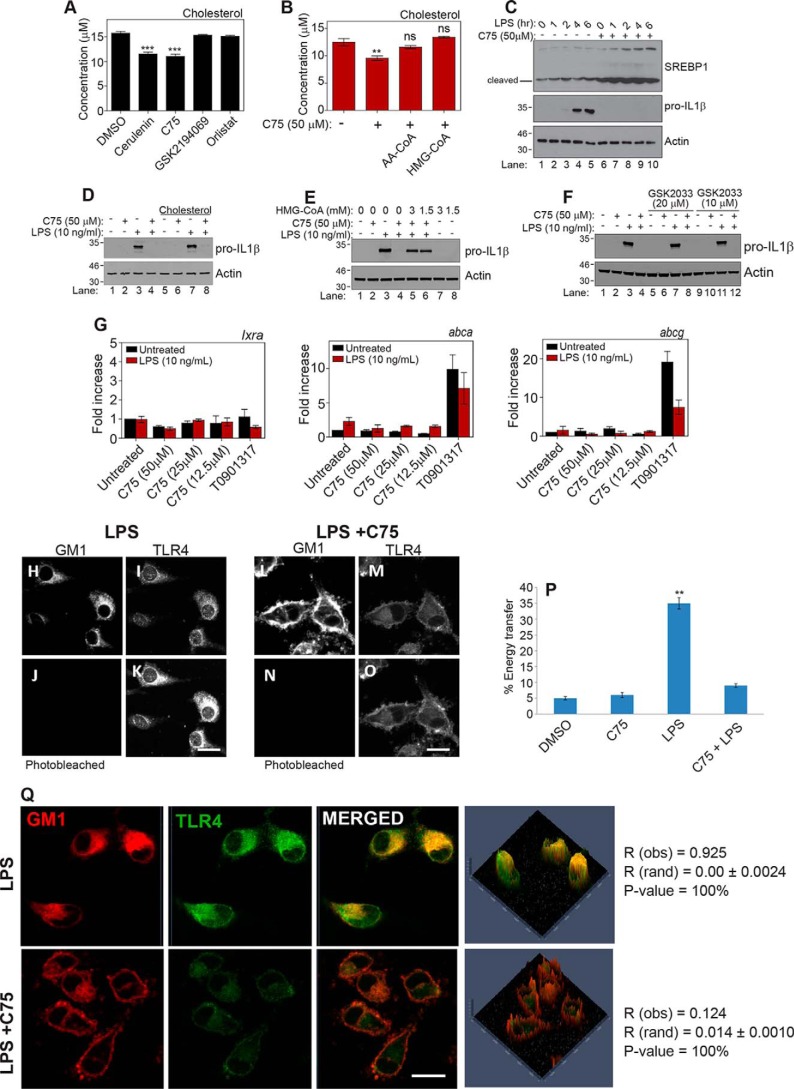
**Fatty acid synthase inhibition decreases cholesterol levels.** *A*, BMDMs were treated with DMSO, cerulenin (50 μm), C75 (50 μm), GSK2194069 (1 μm), and orlistat (50 μm) for 6 h in serum-free medium. Lipids were extracted, and cholesterol was measured using cholesterol oxidase and Amplex red. *B*, BMDMs were treated cultured in serum-free medium alone or serum-free medium containing acetoacetyl-CoA (1 mm) or HMG-CoA (1 mm) for 1 h. Cells were then treated with DMSO or C75 (50 μm) for 6 h. Lipids were extracted, and cholesterol was measured using cholesterol oxidase and Amplex red. *C*, BMDMs were treated with DMSO or C75 (50 μm) for 1 h. Cells were then treated with LPS (10 ng/ml) over a 6-h time course at the indicated times. SREBP1 cleavage and activation were measured by Western blotting. *D*, BMDMs were cultured in medium supplemented with cholesterol (Gibco) for 3 h. Cells were then treated with DMSO or C75 (50 μm) for 1 h, followed by LPS (10 ng/ml) treatment for 4 h. IL1β was measured by Western blotting. *E*, BMDMs were cultured in medium containing the indicated dose of HMG-CoA for 3 h. Cells were then treated with DMSO or C75 (50 μm) for 1 h before treatment with LPS (10 ng/ml) for 4 h. IL1β was measured by Western blotting. *F*, BMDMs were pretreated with the indicated dose of GSK2033 for 1 h. Cells were then treated with DMSO or C75 (50 μm) for 1 h before treatment with LPS (10 ng/ml) for 4 h. IL1β was measured by Western blotting. *G*, BMDMs were treated with the indicated dose of C75 or T0901713 (50 μm) for 1 h. Cells were then treated with LPS (10 ng/ml) for 4 h. mRNA was extracted from whole-cell lysate, and the indicated genes were analyzed by qPCR. *H–O*, BMDMs were treated with DMSO or C75 (50 μm) for 1 h, followed by LPS (10 ng/ml) treatment for 30 min. Energy transfer between TLR4 (*I* and *M*) and GM1 (*H* and *L*) can be detected from the increase in donor fluorescence (*K* and *O*) after acceptor photobleaching (*J* and *N*). The donor (Alexa488) image after acceptor photobleaching (*K* and *O*) demonstrates an increase in fluorescence when cells were stimulated with LPS. FRET was quantified from 10 images of each treatment with 4–5 cells/image. *n* = 3. *P*, energy transfer quantified in response to DMSO, C75, LPS, and LP + C75. *Scale bar*, 10 μm. *Q*, BMDMs were treated with DMSO or C75 (50 μm) for 1 h, followed by LPS (10 ng/ml) treatment for 30 min. *Bar*, 10 μm. The data are representative of three independent experiments. Co-localization coefficients (*R*(obs)) were calculated using Costes' approach. *A* and *B*, data represent mean ± S.E. (*error bars*) (*n* = 3/group): **, *p* < 0.01; ***, *p* < 0.001; ns, not significant, one-way ANOVA.

As palmitate did not rescue the effects of FASN inhibition, this suggested that acetoacetyl-CoA produced by FASN must also have an alternative role that is independent of its role in producing palmitate through FASN. Acetoacetyl-CoA is not only a metabolic intermediate for fatty acid synthesis but also an essential metabolite in cholesterol synthesis. Cholesterol is also a key component of the signaling platforms known as lipid rafts, which are essential for TLR4 signaling (21–23). We hypothesized that FASN may be regulating cholesterol levels, which in turn would alter the lipid raft structure during TLR signaling. This would explain why all tested phosphorylation events of the TLR4 pathway were down-regulated. To investigate whether this link between FASN and cholesterol synthesis existed, we treated BMDMs with the panel of FASN inhibitors and measured cholesterol levels. As shown in Fig. 6*A*, we found that inhibition of FASN did reduce cholesterol levels to a significant degree in BMDMs, but, once again, only inhibitors that inhibited at or above the ketoacyl synthase domain led to a significant reduction in cholesterol levels. This demonstrated that inhibition of FASN also reduced cellular cholesterol levels, providing more evidence that the two pathways are linked. To further investigate the link between FASN and cholesterol synthesis, we next supplemented serum-free medium with acetoacetyl-CoA or a metabolite of cholesterol synthesis, HMG-CoA, followed by FASN inhibition with C75. We found that these metabolites could recover cholesterol levels following FASN inhibition (Fig. 6*B*). A biological hallmark of reduced cellular cholesterol levels is an increase in the cleavage of SREBPs. Fig. 6*C* shows that whereas LPS has little effect on SREBP1 cleavage over a 6-h time course, FASN inhibition does lead to an increase of SREBP1 cleavage (Fig. 6*C*, *compare lanes 6–10* with *lanes 1–5*, respectively). Because SREBP1 is being controlled by SCAP, which is sensitive to cholesterol levels (24, 25), this provides yet another link between FASN and cholesterol synthesis. Exogenous cholesterol was not able to rescue the inhibitory phenotype of C75 (Fig. 6*D*), although it has been shown recently that cholesterol uptake and cholesterol synthesis have differing effects on cholesterol locations and subsequent cellular effects (26). This suggests that cholesterol synthesis and cholesterol uptake are two independent events that do not always compensate for each other (26). To investigate whether cholesterol synthesis could rescue the inhibitory phenotype of FASN inhibition, we supplemented the medium with the cholesterol metabolite, HMG-CoA, and treated BMDMs with LPS in the presence or absence of C75. We found that HMG-CoA could also rescue IL1β production in response to LPS following FASN inhibition (Fig. 6*E*, compare *lanes 5* and *6* with *lane 4*). This is a key finding, as it shows that a cholesterol metabolite can rescue the effects of FASN inhibition and indicates a novel biosynthetic link between FASN and cholesterol synthesis. Interestingly, it has been reported recently that mice specifically lacking FASN in their myeloid cells displayed reduced cellular cholesterol (27). This study suggested that reduction in cholesterol levels upon FASN deletion was due to an increase in LXR-dependent genes increasing cholesterol efflux. We tested this in our system; however, we observed no rescue of IL1β using the LXR antagonist, GSK2033, following treatment with LPS and C75 (Fig. 6*F*, compare *lane 8* with *lane 7* and *lane 12* with *lane 11*). Furthermore, we observed no increase in LXR-dependent genes in C75-treated samples relative to the LXR agonist, T0901317 (Fig. 6*G*; *Lxra*, *Abca*, and *Abcg*). This indicated to us that in our system, the effects seen here are due to a direct biosynthetic link between FASN and cholesterol synthesis rather than the activation of LXRs; however, both observations are likely to contribute to overall cholesterol metabolism in macrophage biology (27). These results place FASN as an important enzyme and regulator of both cholesterol synthesis and TLR signaling during macrophage activation.

### Fatty acid synthase regulation of cholesterol levels is vital to maintenance of lipid rafts and associated inflammatory signaling

Having established the link from FASN to cholesterol synthesis, we next examined lipid rafts, as they contain high levels of cholesterol and are important for TLR and TNF-α signaling (23, 28, 29). We investigated whether inhibition of FASN would have any effects on TLR4 signaling through lipid rafts. As can be seen in Fig. 6 (*H–O*), inhibition of FASN drastically reduces the energy transferred between TLR4 and GM1. Following photobleaching of TLR4 (Fig. 6, *J* and *N* compared with *H* and *L*) we see an increase in fluorescent intensity of GM1 in the LPS-treated macrophages (Fig. 6, *K* compared with *I*). This increase is due to the loss of quenching by the GM1 stain as the TLR4 molecule moved into the lipid raft following LPS stimulation. There is no significant increase in fluorescent intensity of the GM1 stain when the macrophages are treated with C75, suggesting that TLR4 has failed to translocate to the lipid raft (Fig. 6, *O* compared with *M*), and as such there is no quenching of the signal. This energy transfer in stimulated conditions is quantified in Fig. 6*P*. This shows that FASN is essential for maintaining the structure of lipid rafts, most likely via cholesterol synthesis, to facilitate signaling in response external stimuli. We also investigated the role of lipid rafts using colocalization analysis and similarly found that FASN inhibition reduced the co-localization of TLR4 with GM1 ganglioside when compared with the increase in colocalization seen with LPS stimulation (Fig. 6*Q*, *R*(obs) = 0.925 in LPS-treated compared with *R*(obs) = 0.124 in LPS + C75). We also used biochemical isolation of lipid rafts to investigate whether TLR4 translocation was altered by C75. As shown in supporting Fig. 3*A*, we found that TLR4 was present in fractions 1–2 following LPS treatment, indicating that it had translocated to the lipid raft compared with its absence in fractions 1–2 when macrophages had been pretreated with C75. These results demonstrated that inhibition of FASN was preventing TLR4 translocation to the lipid raft.

As CD14 is permanently sequestered in the lipid raft (13, 23), it is likely that the failure of TLR4 translocation to the lipid raft would reduce the signaling in response to LPS stimulation. Taken together, these data identify a biosynthetic link from FASN to cholesterol synthesis in the form of acetoacetyl-CoA with cholesterol synthesis being required for TLR4 signaling.

## Discussion

Here we provide evidence for an unexpected link between the fatty acid synthesis pathway and the cholesterol synthesis pathway with the FASN metabolite, acetoacetyl-CoA, providing the link. We show that FASN is required not only for LPS activation of macrophages but also for macrophage activation by multiple TLR agonists and TNF-α. Our study implicates acetoacetyl-CoA as a metabolite in inflammatory macrophage activation. Our evidence suggests that interfering with acetoacetyl-ACP synthesis by FASN will decrease cholesterol levels and disrupt the lipid raft, resulting in TLR4 failing to translocate to the lipid raft and impairing TLR4 signaling. Whereas there is probably an increase in the proportional amounts of sphingolipids within the lipid raft, leading to brighter GM1 staining, this does not lead to an increase in the transfer of energy to the photobleached TLR4, indicating that the sphingolipids are unlikely to be compensating functionally for cholesterol in TLR4 signaling. It has also been shown that TLR4 contains a cholesterol-binding motif, known as a cholesterol recognition amino acid consensus (CRAC), and this domain is hypothesized to play an important role in translocating TLR4 to the lipid raft for efficient signaling, implying that cholesterol may have a role beyond its role in lipid rafts in our study (30).

Inhibition of FASN resulted in SREBP1 cleavage that is a hallmark of a reduction of cholesterol within the cell. A key piece of evidence for the biosynthetic link between FASN and cholesterol was that HMG-CoA, a metabolite unique to the cholesterol pathway, was able to rescue IL1β production following the suppression caused by FASN inhibition. Overall, our study indicates that along with the switch to glycolysis, an increase in FASN leading to cholesterol production is also required for macrophage activation.

The anti-inflammatory properties of FASN inhibition were not associated with the whole enzyme and its ability to produce palmitate, but rather the anti-inflammatory properties were associated with the ketoacyl synthase domain. The ketoacyl domain of FASN produces acetoacetyl-CoA using the precursor metabolites malonyl-CoA and acetyl-CoA; however, in the cholesterol pathway, acetoacetyl-CoA is synthesized when the thiolase, ACAT, fuses two molecules of acetyl-CoA together. This is interesting, as malonyl-CoA synthesis from acetyl-CoA carboxylase is considered the key step that commits the carbons to fatty acid synthesis. It is also interesting to note that condensation of malonyl-CoA and acetyl-CoA to produce acetoacetyl-CoA is considered a thermodynamically more favorable reaction than acetoacetyl-CoA formation using two acetyl-CoA molecules, as malonyl-CoA contains more free energy from the condensation reaction that forms malonyl-CoA (31). This means that FASN may be a more efficient pathway to alter cellular cholesterol concentrations more rapidly than if the cholesterol pathway alone was used. This is important during inflammation, as cholesterol is a key component of the cell membrane, and the cell membrane plays a key role during macrophage activation. Macrophages not only have to have optimal signaling from the cell membrane (*e.g.* TLR4), but they may also have to alter their membrane drastically to allow for phagocytosis while also ensuring that the membrane stays intact to prevent cell death. This role of cholesterol during macrophage activation may require FASN generated acetoacetyl-CoA to fulfill the cholesterol demands of the cell. Our data are surprising because previous work on FASN implies that there is no mechanism for the release of acetoacetyl-ACP as acetoacetyl-CoA. One possible mechanism is that the malonyl/acetyltransferase domain is a reversible domain, as shown in fatty acid synthesis in bacteria (32). Whereas this domain has been characterized to readily transfer short carbon metabolites to ACP, it is likely that it could work in reverse, similar to in bacteria, to transfer short carbon metabolites back onto CoA.

In a study by Wei *et al.* (27), the role of FASN and macrophages in diet-induced obesity was investigated. They found that a deficiency of FASN specifically in macrophages could help protect against diet-induced insulin resistance and inflammation. Interestingly, they found that macrophages lacking FASN had reduced cholesterol levels that had led to an alteration in the protein content of lipid rafts. This implicates FASN as an important regulator of diet-induced diabetes. Our work provides further support for the role of FASN as a central player in inflammation in more acute conditions, such as infections or sepsis. Whereas chemotherapeutic agents have been developed to target FASN, due to its importance in providing lipid for cell growth in cancer, many of these agents target the ketoacyl-reductase domain, as it is a chemically more favorable target to inhibit. Had similar agents been designed against the ketoacyl-synthase domain, it may have been possible to repurpose these agents as anti-inflammatory agents. It has been also been reported that cerulenin can reduce sterol levels in bacterial cultures (33). The authors report that millimolar levels of cerulenin are required to inhibit HMG-CoA synthase despite only low micromolar levels being required for inhibition of sterol synthesis. Our hypothesis helps to explain this concentration discrepancy, as our data suggest a link between fatty acid synthesis and cholesterol synthesis.

It has been reported that phosphorylation and nitrosylation of fatty acid synthase can regulate its activity, so it is interesting to speculate that, following LPS activation of macrophages, FASN modification can alter the intermediate metabolites produced to better adapt to the situation it requires (34, 35). It would be interesting to see whether FASN or its modified form also plays a role in the regulation of cholesterol levels in other cell types that produce high levels of cholesterol, such as hepatocytes.

In conclusion, we provide a biosynthetic link between fatty acid synthesis and cholesterol synthesis that plays a key role in activation of macrophages and production of IL1β. It has recently been shown that IL1β is an important therapeutic target for risk of a second coronary event in at-risk patients (36). There is a role for both cholesterol and IL1β in this process, because cholesterol crystals activate NLRP3, leading to IL1β production, and activated macrophages are present as foam cells in atherosclerosis (37). Limiting the link between FASN and cholesterol production may have therapeutic potential for the treatment of inflammatory atherosclerosis, where macrophage foam cells play a key role.

## Experimental procedures

### Reagents

LPS used in *in vitro* studies was from *Escherichia coli*, serotype EH100 (Alexis). LPS for *in vivo* trials was from *E. coli*, serotype O55:B5 (Sigma-Aldrich). C75, cerulenin, quercetin, orlistat, GSK2194069, GSK2033, and T09031713 were purchased from Sigma-Aldrich. FASN siRNA 1 (catalogue no. 4390815; assay ID s65865), FASN siRNA 2 (catalogue no. 4390815; assay ID s65867) and scrambled control (catalogue no. 4390843) were purchased from Thermo Fisher Scientific. Heat-killed *Salmonella typhimurium* and *Staphylococcus aureus* were purchased from Invivogen. Antibodies used were anti-IL1β (R&D Systems, AF401-NA), anti-β-actin (Sigma-Aldrich, AC-74), anti-SREBP1 (BD Pharmingen, 557063), anti-FASN (catalogue no. 3180), anti-p65 (catalogue no. 8242), anti-phospho-p65 (catalogue no. 3033), anti-phospho-TAK1 (catalogue no. 4508), anti-phospho-IKKα/β (catalogue no. 2697), anti-tubulin (catalogue no. 5666), and anti-histone H3 (catalogue no. 4499) all from Cell Signaling Technology. Secondary horseradish peroxidase-conjugated anti-mouse IgG, anti-rabbit IgG, and anti-goat IgG were from Jackson ImmunoResearch. Malonyl coenzyme A lithium salt, acetyl coenzyme A sodium salt, butyryl coenzyme A lithium salt, hydroxybutyryl coenzyme A lithium salt, acetoacetyl coenzyme A sodium salt, and hydroxy-3-methylglutaryl coenzyme A sodium salt were purchased from Sigma-Aldrich. CytoTox 96 Non-radioactive Cytotoxicity Assay kit was purchased from Promega. The caveolae/raft isolation kit was purchased from Sigma-Aldrich (CS0750).

### Mouse strains

C57Bl/6 mice were from Harlan U.K. Animals were maintained under specific pathogen-free conditions in line with Irish and European Union regulations. Experiments were approved by local ethical review (Health Products Regulatory Authority) and in accordance with the Animals (Scientific Procedures) Act of 1986 and the GlaxoSmithKline policy on the care, welfare, and treatment of animals.

### Generation of BMDMs

Mice were euthanized in a carbon dioxide chamber, and death was confirmed by cervical dislocation. Bone marrow cells were extracted from the leg bones and differentiated in Dulbecco's modified Eagle's medium (containing 10% fetal calf serum, 1% penicillin/streptomycin, and 20% L929 supernatant) for 6 days, at which time they were counted and replated for experiments.

### Real-time PCR

RNA was isolated using the PureLink RNA minikit (Ambion). cDNA was prepared using by RT-PCR using a high-capacity cDNA reverse transcription kit (Applied Biosystems), according to the manufacturer's instructions. Real-time quantitative PCR (qPCR) was performed on cDNA (25 ng) on a 7500 Fast Real-Time PCR System (Applied Biosystems) using PowerUp SYBR Green Master Mix (Applied Biosystems). The SYBR primer pair sequences were as follows: *Il1b*, 5′-TGGCAACTGTTCCTG-3′ (forward) and 5′-GGAAGCAGCCCTTCATCTTT-3′ (reverse); *Il10*, 5′-AGGCGCTGTCATCGATTT-3′ (forward) and 5′-CACCTTGGTCTTGGAGCTTAT-3′ (reverse); *Tnfa*, 5′-GCCTCTTCTCATTCCTGCTT-3′ (forward) and 5′-TGGGAACTTCTCATCCCTTTG-3′ (reverse); *Adrp*, 5′-AAGAGGCCAAACAAAAGAGCCAGGAGACCA-3′ (forward) and 5′-ACCCTGAATTTTCTGGTTGGCACTGTGCAT-3′ (reverse); *Nqo1*, 5′-GCTGCAGACCTGGTGATATT-3′ (forward) and 5′-ACTCTCTCAAACCAGCCTTT-3′ (reverse); *Rps18*, 5′-GGATGTGAAGGATGGGAAGT-3′ (forward) and 5′-CCCTCTATGGGCTCGAATTT-3′ (reverse).

-Fold changes in expression were calculated using the ΔΔ*Ct* method (38) using mouse Rps18 as the reference gene for normalization. All -fold changes are expressed relative to the untreated control.

### Western blotting

Protein samples from cultured cells were prepared by direct lysis of cells in 2× SDS sample buffer, followed by heating at 95 °C for 7 min. Protein samples were resolved on 8–12% SDS-polyacrylamide gels and were then transferred to nitrocellulose membrane. Membranes were blocked in 5% (w/v) dried milk in Tris-buffered saline/Tween (TBST) for at least 1 h at room temperature. Membranes were incubated with primary antibody, followed by the appropriate horseradish peroxidase–conjugated secondary antibody. They were developed using chemiluminescent (ECL) substrate (Millipore).

### ELISA

Cytokine concentrations in cell supernatants were measured using ELISA Duoset kits for mouse IL10 and TNF-α and IL6, according to the manufacturer's instructions. Cytokine concentrations in serum samples isolated from whole blood were measured using Quantikine ELISA kits for mouse IL1β. Duoset and Quantikine kits were from R&D Systems. Optical density values were measured at a wavelength of 450 nm, using a FLUOstar Optima plate reader (BMG Labtech). Concentrations were calculated using a four-parameter fit curve.

### siRNA transfection of BMDMs

Cells were plated at 1 × 10^6^ cells/ml in 12-well plates overnight. On the day of transfection, the medium was replaced with 500 μl of Dulbecco's modified Eagle's medium without penicillin/streptomycin or FBS. Two Eppendorf microcentrifuges were prepared for each siRNA to be transfected. Opti-MEM (250 μl/well) was added to each tube. RNAimax (add 5 μl/well) was added to one set of tubes, and siRNA (50 nm/well) was added to the second set of tubes. The tubes containing the siRNA were added to the tube with RNAimax, mixed well by pipetting, and incubated for 15 min. The mix (500 μl) was added to each well. 24 h post-transfection, cells were treated as required.

### Confocal microscopy and FRET analysis

BMDM cells were treated as required and washed in PBS following treatments. The cells were fixed with 3.7% paraformaldehyde and stained with an antibody against TLR4 and cholera toxin B subunit CF594 conjugate (Cambridge Biosciences) before imaging on a Carl Zeiss, Inc. LSM510 META confocal microscope (with an Axiovert 200 fluorescent microscope) using a 1.4 numerical aperture ×63 Zeiss objective. The images were analyzed using LSM version 2.5 image analysis software (Carl Zeiss, Inc.). To quantify the degree of co-localization, we used the Costes approach. The Costes approach, Pearson's correlation coefficients, and *p* values were calculated using MBF ImageJ with JACoP (Just Another Colocalisation Plugin). Cells were also analyzed by FRET. As the rate of energy transfer is inversely proportional to the sixth power of the distance between donor and acceptor, the efficiency of energy transfer (*E*) is defined with respect to *r* and *R*_0_, and the characteristic Forster distance is described by the equation, *E* = 1/(1 + (*r*/*R*_0_)6). FRET was measured using a method described previously (23). Analysis was performed on 10 images from each treatment with 4–5 cells/image.

### Lipid raft extraction

RAW cells (1 × 10^8^) were treated with FASN inhibitor, C75 (50 μm), for 1 h. Cells were then treated with LPS (100 ng/ml) for 15 min. Lipid rafts were isolated using the caveolae/raft isolation kit (Sigma-Aldrich) according to the manufacturer's guidelines. After isolation, proteins were concentrated using Strataclean resin (Stratagene), and TLR4 presence or absence in raft fractions was analyzed by Western blotting in different conditions.

## Author contributions

R. G. C. and L. O. conceptualization; R. G. C. and D. C. S. data curation; R. G. C., D. C. S., M. T., and K. T. formal analysis; R. G. C., Z. Z., S. G.-P., S. A., M. T., and K. T. investigation; R. G. C. writing-original draft; Z. Z., S. G.-P., E. L. K., D. C. S., L. K. M., and L. O. writing-review and editing; E. L. K., L. K. M., and L. O. supervision; L. O. funding acquisition.

## Supplementary Material

Supporting Information

## Footnotes

2 The abbreviations used are:

FASN: fatty acid synthase
ACP: acyl-carrier protein
LPS: lipopolysaccharide
TNF: tumor necrosis factor
IL: interleukin
HMG: hydroxymethylglutaryl
SREBP: sterol regulatory element–binding protein
BMDM: bone marrow–derived macrophage
qPCR: quantitative PCR
ANOVA: analysis of variance.
