# Supplementary material for: An unexpected link between fatty acid synthase and cholesterol synthesis in proinflammatory macrophage activation
Source: J Biol Chem. 2018 Feb 20;293(15):5509–21. doi: 10.1074/jbc.RA118.001921 (PMC5900750; doi:10.1074/jbc.RA118.001921)
Supplement: Supporting Information [file supp_293_15_5509__index.html]

An unexpected link between fatty acid synthase and cholesterol synthesis in proinflammatory macrophage activation — FASN driven cholesterol synthesis is needed for TLR activity — An unexpected link between fatty acid synthase and cholesterol synthesis in proinflammatory macrophage activation — FASN-driven cholesterol synthesis is needed for TLR activity — Supporting Information 

# An unexpected link between fatty acid synthase and cholesterol synthesis in proinflammatory macrophage activation

## Supporting Information

- Supporting information - File containing 3 supplemental figures.
