# Supplementary material for: An unexpected link between fatty acid synthase and cholesterol synthesis in proinflammatory macrophage activation
Source: J Biol Chem. 2018 Feb 20;293(15):5509–21. doi: 10.1074/jbc.RA118.001921 (PMC5900750; doi:10.1074/jbc.RA118.001921)
Supplement: Supporting Information [file supp_RA118.001921_135307_1_supp_78005_p4gbzc.docx]

An unexpected link between fatty acid synthase and cholesterol synthesis in proinflammatory macrophage activation

**Richard G. Carroll^1,2^, Zbigniew Zasłona^1^, Silvia Galván-Peña^1,2^, Emma L. Koppe^2^, Daniel C. Sévin^4^, Stefano Angiari^1^, Martha Triantafilou^2,3^, Kathy Triantafilou^2,3^, Louise K. Modis^2^ and Luke A. O’Neill^1,2^ ***

From the ^1^ School of Biochemistry and Immunology, Trinity Biomedical Science Institute, Trinity College, Dublin 2, Ireland; ^2^ Immunology Catalyst, GlaxoSmithKline, Gunnels Wood Road, Stevenage SG1 2NY, UK; ^3^ Institute of Infection and Immunity, School of Medicine, University Hospital of Wales, Cardiff University, Cardiff, UK; ^4^ Cellzome, GlaxoSmithKline, Meyerhofstrasse 1 Heidelberg, 69117, Germany.

**Running title:** FASN driven cholesterol synthesis is needed for TLR activity

Material included:

Supporting figure 1

Supporting figure 2

Supporting figure 3


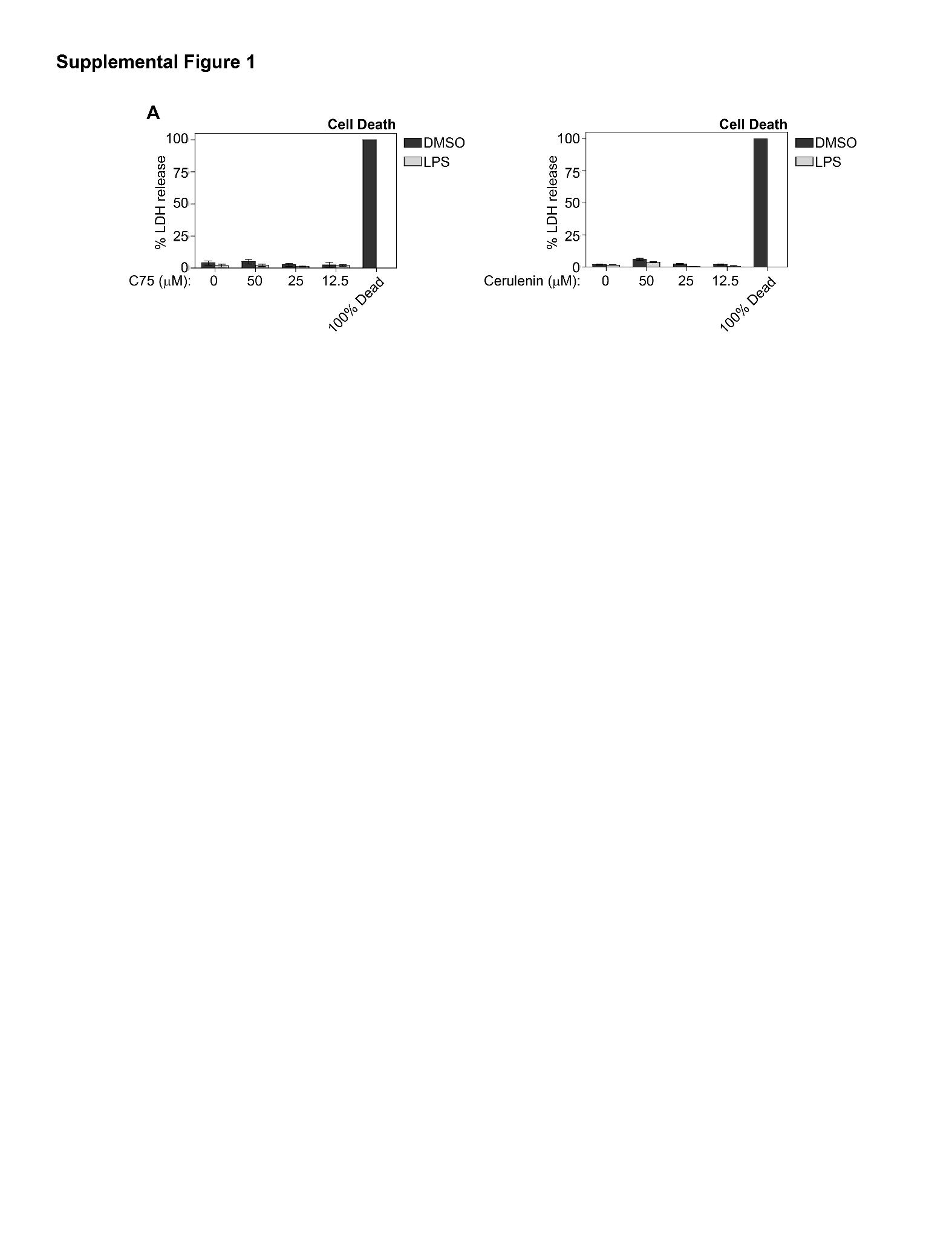


**Supplemental Figure 1. FASN inhibitors do not induce cell death**

(A) BMDMs were treated with DMSO, C75 (50 μM) or Cerulenin (50 μM) for 1 hr. Cells were then treated with LPS (10 ng/mL) for 4 hr. LDH release was analyzed as a read out for cell death. Error bars represent SEM of N=3 experiments.


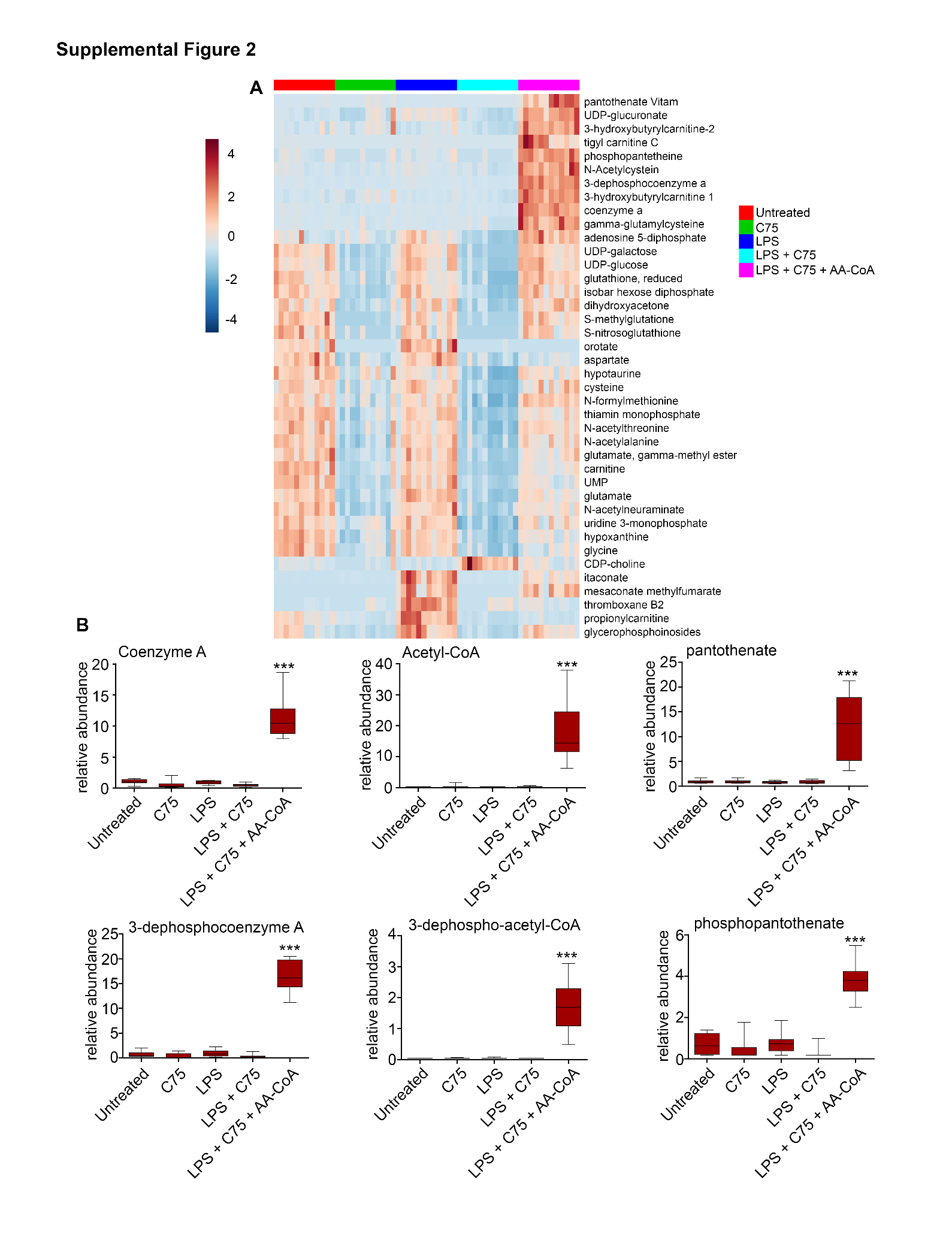


**Supplemental Figure 2. Metabolomic analysis with FASN inhibitors, C75 and acetoacetyl-CoA**

(A) BMDMs were treated as DMSO, C75 (50 μM) and acetoacetyl-CoA (1 mM). Cells were subsequently treated with LPS (10 ng/mL) (1,2). Metabolites were extracted, analyzed and a heat map was generated showing the top 40 changes due to treatment. (B) Plots generated with raw data from metabolomics showing a fold increase in coenzyme A metabolism.


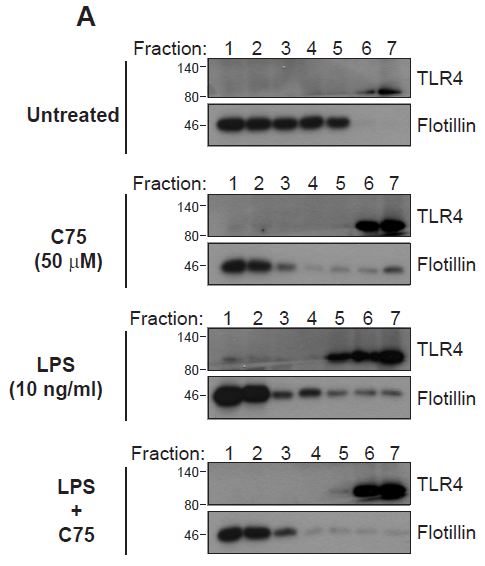


**Supplemental Figure 3. TLR4 translocation to the lipid raft**

(A) Lipid rafts were fractionated optiprep density gradient medium following indicated treatments. Fractions were then tested for the presence of TLR4 and flotillin by western blots.

**Supporting references**

1. Xia, J., and Wishart, D. S. (2016) Using MetaboAnalyst 3.0 for Comprehensive Metabolomics Data Analysis. *Curr Protoc Bioinformatics* **55**, 14 10 11-14 10 91

2. Xia, J., Psychogios, N., Young, N., and Wishart, D. S. (2009) MetaboAnalyst: a web server for metabolomic data analysis and interpretation. *Nucleic Acids Res* **37**, W652-660
